# Individual Uncertainty and the Uncertainty of Science: The Impact of Perceived Conflict and General Self-Efficacy on the Perception of Tentativeness and Credibility of Scientific Information

**DOI:** 10.3389/fpsyg.2015.01859

**Published:** 2015-12-01

**Authors:** Danny Flemming, Insa Feinkohl, Ulrike Cress, Joachim Kimmerle

**Affiliations:** ^1^Leibniz-Institut für Wissensmedien/Knowledge Media Research Center, TübingenGermany; ^2^Max Delbrück Center for Molecular MedicineBerlin, Germany; ^3^Department of Psychology, , University of TübingenTübingen, Germany

**Keywords:** perceived conflict, self-efficacy, critical appraisal, text comprehension, tentativeness, scientific credibility, laboratory experiment

## Abstract

We examined in two empirical studies how situational and personal aspects of uncertainty influence laypeople’s understanding of the uncertainty of scientific information, with focus on the detection of tentativeness and perception of scientific credibility. In the first study (*N* = 48), we investigated the impact of a perceived conflict due to contradicting information as a situational, text-inherent aspect of uncertainty. The aim of the second study (*N* = 61) was to explore the role of general self-efficacy as an intra-personal uncertainty factor. In Study 1, participants read one of two versions of an introductory text in a between-group design. This text provided them with an overview about the neurosurgical procedure of deep brain stimulation (DBS). The text expressed a positive attitude toward DBS in one experimental condition or focused on the negative aspects of this method in the other condition. Then participants in both conditions read the same text that dealt with a study about DBS as experimental treatment in a small sample of patients with major depression. Perceived conflict between the two texts was found to increase the perception of tentativeness and to decrease the perception of scientific credibility, implicating that text-inherent aspects have significant effects on critical appraisal. The results of Study 2 demonstrated that participants with higher general self-efficacy detected the tentativeness to a lesser degree and assumed a higher level of scientific credibility, indicating a more naïve understanding of scientific information. This appears to be contradictory to large parts of previous findings that showed positive effects of high self-efficacy on learning. Both studies showed that perceived tentativeness and perceived scientific credibility of medical information contradicted each other. We conclude that there is a need for supporting laypeople in understanding the uncertainty of scientific information and that scientific writers should consider how to present scientific results when compiling pertinent texts.

## Introduction

When searching for information, laypeople traditionally expect scientific research to provide guiding knowledge (Niaz, 2010). In a variety of situations, laypeople are confronted with scientific information they have to assess and evaluate (Scharrer et al., 2012; Bromme et al., 2014). People frequently feel a need to be better informed, but scientific issues are usually complex, and often they are controversial even among professional scientists. How do people without domain expertise deal with the complexity and *uncertainty* inherent in scientific information as it is presented in texts?

As a matter of fact uncertainty and *tentativeness* are characteristic for empirical results (Popper, 1968; Bromme and Goldman, 2014; Kimmerle et al., 2015a). Controversies are a daily occurrence in empirical sciences (Friedman et al., 1999). “Instead of solid knowledge, we should get used to the notion of tentative information” (Ioannidis, 2006, p. 575). Accordingly, laypeople should also understand and comprehend the tentativeness of findings in scientific research. This means, for instance, to be aware that research findings are not to be understood as everlasting absolute truth but are more or less valid explanations that scientists deduct from empirical studies that can have different levels of quality and underlie several limitations (Lederman and O’Malley, 1990; Jensen, 2008). Understanding tentativeness also means to comprehend that these findings may contradict each other or become obsolete when more reliable findings occur (see Sinatra et al., 2014).

So far, however, it is largely unclear to what extent laypeople are capable of such elaborate assessment processes. Are they aware of the tentativeness when they read about new and yet uncertain empirical findings? At the same time, of course, laypeople should show at least some degree of trust in reputable scientific research. This perceived scientific *credibility* includes two aspects: the belief that the research is actually science-based and that it is reliable and trustable. It is quite plausible, however, that it is exactly the perception and understanding of the tentativeness of research findings that might undermine the perceived scientific credibility of these findings (Kortenkamp and Basten, 2015). Accordingly, we hypothesize that:

(H1) Perceived tentativeness and perceived scientific credibility of scientific information will contradict each other.

It is particularly relevant to examine how tentativeness and credibility as two aspects of the *uncertainty of science* are related to people’s *individual aspects of uncertainty*, which may arise either in a given situation (e.g., from informational contradictions) or result from personal dispositions. Informational contradictions occur quite easily because, very often, people use more than one source for evaluating scientific information (Brossard, 2013; Maier and Richter, 2014). This may lead to a situation, for example, where they encounter positive and optimistic information about a medical treatment from one text and negative or critical assessment of the same treatment from another source (see Rogers and Gould, 2015). This constellation is supposed to result in some sort of cognitive conflict in the recipient (see Piaget, 1977; Limón, 2001; Kimmerle et al., 2011, 2015b). Such a perceived conflict, in turn, may have an impact both on the understanding of the tentativeness of scientific research findings and on the perceived scientific credibility of this research. Previous research has shown that conflicting information can lead to confusion and a skeptical attitude toward science (Covello and Peters, 2002; Vardeman and Aldoory, 2008; Kortenkamp and Basten, 2015). Blankenship and Holtgraves (2005) found that written messages that mentioned the tentativeness of scientific findings provoked more critical attitudes toward this message. Chinn and Brewer (1993, 1998) showed that perceived conflict due to anomalous data can lead to reactions such as ignoring, rejecting, or questioning the validity of these data. Since a higher perceived conflict needs rational elaboration to be resolved and enable conceptual change (Posner et al., 1982; Limón, 2001), a cognitive conflict may also activate laypeople to engage in even deeper processing of information and arguments, which supports them in conducting appropriate critical appraisal (Sinatra and Dole, 1998). Such critical appraisal applies to the veracity and persuasiveness of scientific claims (Guyatt et al., 2008; Scharrer et al., 2012) and should, accordingly, have an impact on perceived tentativeness and credibility of research findings. In light of these considerations, we hypothesize that:

(H2) Contradicting information will lead to a perceived conflict,
(H3) Higher perceived conflict will lead to a stronger perception of tentativeness, and
(H4) Higher perceived conflict will lead to a decreased perception of scientific credibility.

While perceived conflict represents an uncertainty factor directly induced by situational aspects (here: contradictions between information from different texts), the perception and evaluation of tentativeness and scientific credibility may also be influenced by personal aspects such as trait variables. One factor that has an impact on whether people are confident or experience uncertainty is their sense of *self-efficacy.* General self-efficacy is the individual conviction to be able to cope with critical situations (Bandura, 1997).

On the one hand, it has been shown that “individuals with higher self-efficacy are capable of completing a challenging task and are likely to engage in the challenge” (Lo et al., 2013, p. 56). So one might assume that a high level of self-efficacy would come along with improved processes of attention and elaboration. This could result in better information processing, which might also apply to the perception of tentativeness and credibility of scientific information.

On the other hand, previous findings suggest that one’s critical appraisal can be impaired by personal overestimation of the own cognitive ability (Ackerman et al., 2002). Such an overestimation could result from a high level of general self-efficacy since general self-efficacy leads to a more optimistic estimation of one’s own competences and abilities (Caprara et al., 2011; Bandura, 2012; see Pintrich et al., 1993). Karademas et al. (2007) even showed that high self-efficacy and profuse optimism may cause biased information processing. This is supported by a recent meta-analysis that scrutinized influencing factors on individuals’ overestimation of their own abilities (Zell and Krizan, 2014).

Because overestimation is particularly likely to happen in domains where an individual does not have sufficient competencies (Ehrlinger et al., 2008), we expect laypeople to be affected by this effect. In scientific contexts laypeople have low abilities for critical appraisal (Chinn, 2011). This means that laypeople with a high level of general self-efficacy would be less able to recognize the tentativeness of scientific research findings appropriately, while at the same time would overrate their scientific credibility. Accordingly, we hypothesize that:

(H5) Higher general self-efficacy will be associated with a weaker perception of tentativeness and
(H6) Higher general self-efficacy will be associated with an enhanced perception of scientific credibility.

We tested H2-4 and H5-6 separately in two laboratory studies and examined H1 in both studies.

## Study 1

### Materials and Methods

#### Study Design

We conducted an experimental study using a between-group design. Participants were randomly allocated into one of two experimental conditions, confronting them either with two contradicting or two non-contradicting sources of information. Outcome variables were *perceived conflict, perceived tentativeness*, and *perceived scientific credibility.* Additionally, several demographic measures (sex, age, final school grade) were collected to control for possible confounders.

#### Participants

The sample consisted of 48 participants of 19–71 years recruited via a University-wide recruiting panel. This panel is composed of volunteers from the local population and includes people with wide demographic backgrounds but with university students as the major audience. Those volunteers have agreed to receive e-mail invitations for participation in laboratory studies to which they may enroll if they are interested. All participants in this study were German native speakers, stated to have no prior knowledge about the medical domain used in our material, gave full written informed consent, and were naïve to the purposes of the study. Students of medicine and psychology were not admitted to participation. **Table 1** shows the demographic features of this sample.

**Table 1:** Sample demographics of Study 1.

Sex	33 Women (68.75%)	15 Men (31.25%)	
Age	*M* = 28.94	*SD* = 11.29	Range: 19–71
Final school grade	*M* = 2.05	*SD* = 0.58	Range: 1.0–3.4

*Note for school grade: 1.0 = best, 4.0 = worst.*

Participation took about 60 min and was compensated with 8 Euros. Ethical approval was obtained from the institutional board of ethics.

#### Procedure

After giving informed consent, participants read an *introductory text* about a medical topic. The topic chosen here was the neurosurgical procedure of deep brain stimulation (DBS). This text came in two different versions—depending on the experimental condition. One text version expressed a rather positive attitude toward DBS and one focused on the negative aspects of this procedure (see next section for details). After that, the participants in both conditions read the same *target text* about the experimental application of DBS as a treatment for patients suffering from intractable major depression. The results presented in this second text were largely positive so that the participants in one condition had to deal with two contradicting sources of information while the participants in the other condition had to deal with non-contradicting sources of information. This experimental treatment was followed by a manipulation check and three questionnaires that measured the outcome variables. Concluding, demographic information was collected.

#### Instruments and Material

##### Introductory text

The first text participants had to read gave an overview about DBS in general and as a therapy for Parkinson’s disease in particular. This text consisted of 543 (positive version) or 607 (negative version) words, respectively. Both texts outlined the procedure and the functional principle of DBS, which consists of implanting electrodes in particular brain areas in order to stimulate or inhibit functional systems via slight electrical tension. This does not cure the ultimate causes of psycho-motoric diseases but results in diminished motoric symptoms permitting patients to regain substantial quality of life and independence. Despite the possible benefits in several application areas (e.g., Parkinson’s disease, essential tremor; Garonzik et al., 2013), experimental use of DBS in other fields such as depression or anxiety disorders is a controversial issue since there is lack of empirical evidence (e.g., Lozano et al., 2012) and several ethical concerns (Clausen, 2009, 2011).

The positive version of the text mainly focused on chances and success of DBS (e.g., improvement of walking and balance dysfunctions in Parkinson’s patients), while the negative version focused on risks and possible side-effects (such as complications that might occur during or after surgery). Nevertheless, the communicated facts about DBS were the same in both conditions and all pieces of information were scientifically correct.

##### Target text

All participants read the same text that dealt with a single, small-sample exploratory study on DBS as a new treatment for intractable major depression. This target text consisted of 812 words. It portrayed the study results in a positive frame (e.g., “The brain pacemaker that was implemented alleviated symptoms of depression in six of seven patients”). But it also provided references to the tentativeness of the study findings (e.g., “‘We definitely need larger samples to ascertain that we actually found a more effective solution than in earlier attempts’, neurosurgeon Karl Kiening … pronounces”; see e.g., Kiening and Sartorius, 2013). Thus, the article delivered neutral facts from a slightly optimistic point of view, but also highlighted the limitations of the study (e.g., lack of a control group, no long-term observation, etc.).

##### Questionnaires

After reading the two texts, participants were asked to indicate their *perceived conflict* between the texts by rating their agreement to five statements on a 7-point Likert scale. The sum of all item scores (some reversed) resulted in a possible score range of 5–35, with higher values indicating higher perceived conflict. The items of this scale are presented in **Table 2**.

**Table 2:** Perceived conflict questionnaire.

Number	Item
(1)	The texts contradict each other.
(2)	With regard to content, the texts complement one another very well.^∗^
(3)	Overall, the texts provide a balanced picture of the topic.^∗^
(4)	After reading the texts, I find it hard to deliver a concluding judgment on DBS.
(5)	The texts concur in their basic message.^∗^

*^∗^Reversed items.*

*Perceived tentativeness* of the findings presented in the target text was measured by a 7-point Likert scale as well. It consisted of five items yielding a possible score range of 5–35, with higher values indicating higher perceived tentativeness. **Table 3** shows all items of this questionnaire.

**Table 3:** Perceived tentativeness questionnaire.

Number	Item
(1)	The results of the study are not very definite.
(2)	Our knowledge about the application of DBS for treating depression is not complete yet.
(3)	The study is conclusive.^∗^
(4)	Correct conclusions were drawn from the results.^∗^
(5)	The study provides a stable basis to decide about future application or non-application of DBS in depression.^∗^

*^∗^Reversed items.*

Participants had to rate three items asking for the *perceived scientific credibility* of the target text on a 7-point Likert-scale, resulting in a possible score range of 3–21. Higher values represent higher perceived scientific credibility. **Table 4** presents all items of this questionnaire.

**Table 4:** Perceived scientific credibility questionnaire.

Number	Item
(1)	How science-based was the text?
(2)	How credible was the text?
(3)	As how scientifically credible would an expert rate the text in your opinion?

### Results

**Table 5** shows descriptive statistics for the three outcome variables.

**Table 5:** Descriptive statistics of variables in Study 1.

Scale	Items	Possible range	Mean	*SD*
Perceived conflict	5	5–35	16.62	5.86
Perceived tentativeness	5	5–35	22.52	4.07
Perceived scientific credibility	3	3–21	11.96	3.86

To test the hypothesis that perceived tentativeness and perceived scientific credibility would contradict each other (H1), we calculated Spearman’s correlation coefficient between those dependent variables. As expected, we found a significant negative relationship: *r* = -0.421; *p* = 0.003.

In (H2) we expected that contradicting sources of information would lead to a perceived conflict. We tested this hypothesis by conducting a one-sided *t*-test. As assumed we found that after reading the target text the participants who first read the negative version of the introductory text perceived a higher conflict between the two texts (18.04 ± 5.37) than the participants who read the positive version of the introductory text (14.95 ± 6.10), [*t*_(48)_ = 1.863; *p* = 0.035].

We also expected that higher perceived conflict would lead to a stronger perception of tentativeness (H3) and to a decreased perception of scientific credibility (H4). We tested both hypotheses via linear regression models. As expected, the linear regression model on perceived tentativeness as dependent variable revealed a significant positive association with perceived conflict (standardized β = 0.513; *p* < 0.001). Thus, the higher the perceived conflict between the introductory text and the target text, the better people were able to detect tentativeness of findings described in the latter.

As hypothesized, the linear regression model on perceived scientific credibility as dependent variable revealed a significant negative impact of perceived conflict (standardized β = -0.374; *p* = 0.012). Hence, the higher the perceived conflict between the introductory text and the target text, the lower people rated the scientific credibility of the findings described in the second text.

The results of both models did not change substantially if controlled for sex, age, and final school grade. Nevertheless, we cannot exclude possible influences of individual trait variables that may also have an impact on measures of critical appraisal. Thus, we conducted another study that focused on the impact of a potentially relevant trait variable.

## Study 2

### Materials and Methods

#### Study Design

In this laboratory study all participants read a target text about DBS. As a trait variable we measured participants’ *general self-efficacy*. Dependent variables were *perceived tentativeness* and *perceived scientific credibility*. To control for possible confounders, we also collected demographic data (sex, age, final school grade).

#### Participants

From the same panel as described in Study 1, we recruited 61 students from 18 to 35 years who all were native speakers, stated to have no prior knowledge of DBS, gave full written informed consent, and were reimbursed for their participation. Students of medicine and psychology were excluded from participation. Volunteers who had already taken part in Study 1 were not admitted to participate. **Table 6** shows the demographic features of this sample.

**Table 6:** Sample demographics of Study 2.

Sex	48 Women (78.69%)	13 Men (21.31%)	
Age	*M* = 23.34	*SD* = 3.59	Range: 18–34
Final school grade	*M* = 2.03	*SD* = 0.64	Range: 1.0–3.5

*Note for school grade: 1.0 = best, 4.0 = worst.*

Participation took about 45 min and was compensated with 6 Euros. Ethical approval was obtained from the institutional board of ethics.

#### Procedure

After giving informed consent, all participants read a text about the experimental application of DBS as treatment for patients suffering from major depression. Then they filled in a set of questionnaires (*general self-efficacy*, *perceived tentativeness*, and *perceived scientific credibility*). Finally, participants were asked for demographic information.

#### Instruments and Material

##### Target text

We used the same text as in Study 1, only slightly adapted and complemented by some subheadings for better readability, resulting in 832 words.

##### Questionnaires

Administered questionnaires were basically the same as in Study 1. While the questionnaire for *perceived scientific credibility* was identical to that in Study 1 (see **Table 4**), the questionnaire for *perceived tentativeness* was altered to improve accuracy (regarding concrete reference to the study reported in the target text) and to address more facets of tentativeness (see Bromme and Goldman, 2014). The revised form consisted of six items yielding a possible score range of 6–42, with higher values indicating higher perceived tentativeness. **Table 7** shows the revised questionnaire.

**Table 7:** Perceived tentativeness questionnaire – revised form.

Number	Item
(1)	The results of the study are not very definite.
(2)	After this study, our knowledge about the application of DBS for treating depression is not complete yet.
(3)	The study is conclusive.^∗^
(4)	The findings are reliable.^∗^
(5)	The study provides a stable basis to decide about future application or non-application of DBS in depression.^∗^
(6)	The results of the study should be viewed as tentative.

*^∗^Reversed items.*

*General self-efficacy* was obtained using the *General Self-Efficacy Scale (GSE)* by Schwarzer and Jerusalem (1995). This questionnaire consists of 10 statements that participants had to rate regarding their agreement or disagreement on a 4-point scale. Summing up the responses to all ten items resulted in a sum score ranging from 10 to 40. The whole scale is depicted in **Table 8**.

**Table 8:** General self-efficacy scale (Schwarzer and Jerusalem, 1995).

Number	Item
(1)	I can always manage to solve difficult problems if I try hard enough.
(2)	If someone opposes me, I can find the means and ways to get what I want.
(3)	It is easy for me to stick to my aims and accomplish my goals.
(4)	I am confident that I could deal efficiently with unexpected events.
(5)	Thanks to my resourcefulness, I know how to handle unforeseen situations.
(6)	I can solve most problems if I invest the necessary effort.
(7)	I can remain calm when facing difficulties because I can rely on my coping abilities.
(8)	When I am confronted with a problem, I can usually find several solutions.
(9)	If I am in trouble, I can usually think of a solution.
(10)	I can usually handle whatever comes my way.

### Results

**Table 9** shows descriptive statistics for *general self-efficacy*, *perceived tentativeness*, and *perceived scientific credibility*.

**Table 9:** Descriptive statistics of variables in Study 2.

Scale	Items	Possible range	Mean	*SD*
General self-efficacy	10	10–40	30.18	4.46
Perceived tentativeness	6	6–42	28.84	4.76
Perceived scientific credibility	3	3–21	11.70	3.45

Like in Study 1, we expected that perceived tentativeness and perceived scientific credibility would contradict each other (H1). Again, we calculated Spearman’s correlation coefficient between perceived tentativeness and perceived scientific credibility to test this hypothesis. As expected, we found a negative relationship between those variables: *r* = -0.389; *p* = 0.002.

We further assumed that higher general self-efficacy would be associated with a weaker perception of tentativeness (H5) and with an enhanced perception of scientific credibility (H6). We tested these hypotheses using linear regression models.

As hypothesized, linear regression modeling revealed a significant negative association between general self-efficacy and perceived tentativeness. The higher participants’ general self-efficacy, the less they were able to detect the tentativeness of findings described in the target text (standardized β = -0.289; *p* = 0.024).

As assumed, the linear regression model on perceived scientific credibility showed a significant association with general self-efficacy. The higher participants’ general self-efficacy, the higher they rated the scientific credibility of the findings described in the target text (standardized β = 0.406; *p* = 0.001).

The results of both models did not change substantially if controlled for sex, age, and final school grade.

## Discussion

In Study 1, we examined whether the processing of contradicting texts sources had an impact on laypeople’s perception of conflict and how this influenced their critical appraisal. As assumed, we found that the processing of contradicting sources resulted in higher perceived conflict, which, in turn, lead to a stronger perception of tentativeness as well as to a decreased perception of scientific credibility of a medical study reported in the second text. Accordingly, perception of tentativeness and perception of scientific credibility were negatively correlated.

We could show in Study 1 that external, that is, text-inherent factors had a significant impact on critical appraisal. Although the target text was totally identical in both experimental conditions, the mere perception of a conflict between the general message of this text and a prior introductory text made a decisive difference in how participants processed and assessed the second text. They were more critical the higher they perceived the conflict between the two texts, which became apparent in a better detection of tentativeness and the lower ratings for perceived scientific credibility. This is in line with the aforementioned findings of Covello and Peters (2002) as well as Vardeman and Aldoory (2008) that conflicting information can lead to a more skeptical attitude toward science and medicine. While a better detection of tentativeness is probably preferable, increased skepticism against science itself is not a goal of science communication. It should be an aim of science education to explain that salience of tentativeness is not necessarily a clue for unreliable or untrustworthy science, but rather for a correct and precise consideration of the limitations and boundaries, which is an eminent element of accuracy and quality in science.

Due to the fact that there are no established tools yet to measure our dependent variables, we had to develop our own scales for perceived conflict, perceived tentativeness, and perception of scientific credibility. Although successfully tested in a material test and a prior study, external validity cannot be ultimately guaranteed. In addition, we do not know whether the influence of conflicting information also works outside the laboratory, where the participants were focused and instructed to read the texts one after another. One may assume that in real-life information search, occurrence of cognitive conflict is less likely than in our laboratory situation because laypeople would potentially tend to avoid information that seems to be contradictory to their prior knowledge or attitude (Chinn and Brewer, 1998).

In Study 2, we tested how general self-efficacy as an individual trait variable affected laypeople’s ratings of tentativeness and scientific credibility when reading science-related texts. We could replicate our finding from Study 1 that perception of tentativeness and perceived scientific credibility are opposed to each other. Furthermore, we found a significant negative association between general self-efficacy and perceived tentativeness as well as a significant positive association between general self-efficacy and perceived scientific credibility.

Due to the fact that self-efficacy was not experimentally manipulated but measured as a trait variable, we have to be careful in interpreting these findings with respect to their causality. Nevertheless, since we can assume that general self-efficacy is a rather stable trait variable and changes only over longer time periods (Bandura, 2012; Ouweneel et al., 2013), it seems plausible that it was the inter-personal differences in general self-efficacy that predicted parts of the variance in perceived tentativeness and perceived scientific credibility, not the other way round.

Our findings are limited by the fact that our samples consisted mainly of university students who may be more qualified to rate the tentativeness and scientific credibility of information from scientific texts than the general population. In addition, it is not entirely clear how the effect of general self-efficacy on critical appraisal works in real-life-conditions outside the laboratory.

Taken together, the results of both studies show that when laypeople read texts that contain scientific information, their perception of tentativeness and the perceived scientific credibility of the information are diametrically opposed. It seems that the better they are able to detect and understand that the findings mentioned in the text have to be seen as tentative and fragile and as a result of the scientists’ observation and interpretation of one single study, the more they doubt the scientific trustworthiness of the text that reports these findings. On the one hand, it seems preferable when laypeople learn to view scientific texts with adequate skepticism and form their own opinion, integrating all positive and negative findings and results about a certain scientific topic. On the other hand, one could also interpret these results in the way that laypeople seem to have more trust toward scientific results, the more definite and unquestioningly they are presented (Kimmerle et al., 2013). From the eyes of a scientific expert, however, such reduction of complexity would mean a loss of accuracy, and, as a consequence, a loss of scientific credibility (Bientzle et al., 2013). Thus, texts that are scientific for experts may appear to be less “scientific” to laypeople. There is obviously eminent need for training laypeople’s capabilities for adequate appraisal. Moreover, experts and science journalists should be aware of this dilemma and consider in detail which phrases to use and how to present scientific results when compiling pertinent texts. Our results also show that laypeople’s perception of tentativeness and perceived scientific credibility are affected by both situational and personal aspects. The influence of text-inherent variables can be taken into account and adequately addressed by scientific writers, in terms of whether and how to state out conflicts within the topic or whether and how to make tentativeness salient in a text (Kimmerle et al., 2015a). This requires awareness about the effect of these factors amongst science journalists and other authors who deal with scientific information.

However, personal trait variables like general self-efficacy cannot be influenced by text composition. But at least, authors should be aware that appraisal of the results mentioned in their texts can highly differ due to interpersonal differences. Thus, it is all the more important that writers take text-inherent factors into account in order to support laypeople in understanding the tentativeness of scientific information as far as possible. Another practically relevant question is how those people with a high level of general self-efficacy—who appear to be vulnerable to underestimating the tentativeness of scientific information—could be prevented from a too naïve perception of scientific texts. This issue needs to be addressed in future studies.

What our results cannot reveal is what interpersonal differences in perception of tentativeness and perceived scientific credibility may implicate. There are well-examined positive effects of high self-efficacy on learning (e.g., Pintrich and de Groot, 1990; Linnenbrink and Pintrich, 2003), so high self-efficacy has been shown to be a beneficial trait from an educational point of view. Apparently in contradiction to this, it seems that although high self-efficacy may support learning, it does not support critical appraisal, but comes along with a more naïve reception of information. This contradiction should be examined in future research as well. It would be a desirable educational goal to support laypeople in learning from a scientific text, while at the same time supporting them in critically evaluating its content.

While our results show that—under certain conditions—when asked by a questionnaire, laypeople are able to assess tentativeness in texts that contain scientific information, little is known about how perceived tentativeness influences their opinion formation, attitude and, in consequence, judgements about health-related issues as whether to undergo a certain treatment or not (see Bientzle et al., 2015; Fissler et al., 2015). These questions should also be addressed in further studies.

## Conflict of Interest Statement

The authors declare that the research was conducted in the absence of any commercial or financial relationships that could be construed as a potential conflict of interest. The research reported here was funded by a grant from the German Federal Ministry of Education and Research. This sponsor did not exert influence on the content of this research in any way.
